# Chicago Medical Society, November 4th

**Published:** 1885-12

**Authors:** 


### SOCIETY REPORTS.

---

CHICAGO MEDICAL SOCIETY.

*Stated Meeting, November 4th, 1885.*—The PRESIDENT, C. T. PARKES, M. D., in the chair.

NERVOUS PAROXYSM, OR SYNCOPE, was the subject of a paper by DR. H. T. BYFORD. He read the report of two fatal cases, which had not occurred in his own practice, and traced the cause of death to strangulation by medicine given during the paroxysms. The third case reported occurred in his own practice and recovered, no attempt at internal medication having been made during the paroxysms. After a recital of the cases, the author proceeded to describe this hitherto imperfectly recognized form of nervous paroxysm, which, in the books and in practice, is often confounded with true syncope, hysteria and other conditions, but which is in reality more in the form of convulsions.

In the cases which he had treated, the most common exciting causes lay in the digestive system, but he thought subsequent observations would demonstrate that the exciting causes might also be due to some disturbance of the cutaneous, osseous, sexual or urinary systems. The predisposing causes are debility, plethora, sudden change of habits, severe strains upon vital forces, parturition, functional or organic diseases, etc.

The author tabulated the symptoms occurring in true syncope and nervous paroxysm, as follows:

| TRUE SYNCOPE. | NERVOUS PAROXYSM. |
| --- | --- |
| 1. Often preceded by a feeling of oppression about the epigastrium, or nausea and general malaise. Patients feel the attack coming on. | 1. Often preceded by nervous irritation, heaviness about the stomach, and frequent desire to take a deep inspiration (gaping); sometimes by a feeling of nervous exaltation, or equanimity greater than usual. Onset is usually sudden. |
| 2. Pallor, vacant expression of countenance, and mental calm in the beginning, with dullness of special and general sensation. | 2. Pallor, anxious countenance, mental distress in the beginning, with acuteness of special and general sensation. |
| 3. Muscular relaxation. | 3. Muscular rigidity or activity. |
| 4. Dyspnœa, if any, not distressful—objective rather than subjective. | 4. Dyspnœa distressful — more subjective than objective. |
| 5. Unconsciousness absolute. | 5. Seldom absolute unconsciousness, mental confusion or incapacity. |
| 6. Unless fatal, attack passes away rapidly, especially if patient in recumbent position. | 6. Passes away gradually, more rapidly if head and shoulders be elevated. |
| 7. Temperature normal or below (unless elevated by disease). | 7. Temperature normal or above. |
| 8. Seldom recurs if head be kept low. | 8. Usually recurs, or threatens to recur shortly, even if patient lie down. |
| 9. Heart's action greatly depressed. | 9. Heart's action irregular, spasmodic, and only moderately depressed. |
| 10. Seldom very disagreeable recollections of distress during the attack. | 10. Very disagreeable recollections of distress during the paroxysms. |
| 11. Stimulants afford relief. | 11. Stimulants aggravate. |

Hysteria, uræmia, the action of remedies. etc., may complicate and obscure the symptoms in any given case. The treatment calls for counter-irritation, cool air, cold drinks, massage, antispasmodics, for the paroxysms; afterwards, a removal of the cause, if possible, and measures for the restoration of good health.

DISCUSSION.

PROFESSON J. H. ETHERIDGE: I would like to ask DR. BYFORD what he would think of the use of hypodermic injections of digitalis under such circumstances; to ask if any one has ever used it in cases of acute syncope; and why it would not be a good thing to make use of?

DR. H. T. BYFORD: In these attacks I believe digitalis would perhaps hardly be the remedy, for the condition is one of reflex irritation, I think. I think it is a case of spasmodic contraction of the heart, and that any nervous symptoms are the result of a contraction of the arterioles of the brain rather than a falling off in the force of the circulation, and that stimulants, such as digitalis, would be liable to do harm. This class of cases I have considered really attacks of convulsions, in which consciousness is not lost, if such a thing can be admitted. In these cases consciousness is not lost; sometimes there is a partial loss of consciousness, spasmodic contraction of the diaphragm, the breath almost entirely cut off, and I suppose the same spasmodic condition of the heart; and sometimes, and in some of the cases referred to, there was rigidity of the muscles; one patient tried to throw herself out of bed. In real syncope the attacks involve loss of consciousness, and relaxation of the muscles. Dr. G. NEWKIRK has informed me that he formerly treated cases of malaria wherein the onset was marked by symptoms such as have been described. These cases, occurring in a malarial district, were characterized by a foul stomach, due to the presence of acrid ingesta. Free vomiting, spontaneous or induced, nearly always gave relief.

PALATABLE THERAPEUTICS was the subject discussed in a paper read by Dr. FRANKLIN H. MARTIN. He exhibited specimens of drugs manufactured in the form of pills, capsules, granular salts, elixirs, et cetera, either tasteless or palatable. In his paper, DR. MARTIN claimed that many of the greatest improvements in therapeutics have been gleaned from the field of charlatanism. Electricity, massage and the "Swedish movement" have been wrested from this domain, and placed upon a legitimate and scientific basis. The author claimed that homœopathic medication had three valuable characteristics, palatability, harmlessness and inexpensiveness to the patients. This combination, absolutely without medical merit, accounts for the so-called success of homœopathy. If we can secure palatability to our remedies, we will do much to overcome the popular aversion to our medicinal preparations. Since 1817, when morphia was first isolated as an alkaloid, a great advance has been made in discovering the active principles of drugs. Of the alkaloids there have now been discovered twenty-two, with actions similar to the parent drugs. They are manufacfactured in pill form and are more easy of administration than the disagreeable preparations from the crude drugs. Twenty glucosides have been similarly isolated and manufactured. While this branch of pharmaceutical chemistry is yet in its infancy we can confidently look forward to the day when the active principles of all the organic drugs will have been discovered, isolated and manufactured into palatable preparations. To-day, the salts of various metals, solid extracts, the bromides and iodides, chloral hydrate and the oils can all be administered in capsules. DR. MARTIN claimed to be the first to suggest the enclosing in capsules solutions of chloral in oil. We can summarize the means by which we can make medicines palatable, as follows: The administration of alkaloids, solid extracts, crude drugs of small bulk, and various salts in capsules or gelatine or sugar-coated pills; the administration of glucosides and neutral principles in gelatine or sugar-coated granules; the administration of tasteless liquids in water; the administration of oils, oleo-resins, oleates and drugs soluble in oil in soft elastic gelatine capsules; and the administration of medicines by the hypodermic syringe, suppositories and inunctions.

DISCUSSION.

DR. J. J. M. ANGEAR: The object of the paper is certainly commendable. I think the latter part of the paper is valuable, where is urged upon us the necessity of discarding these nauseating remedies and availing ourselves of the science of chemistry, and we should take the admonition to ourselves. I think we are injuring ourselves and our profession by prescribing such crude material when we might avail ourselves of more modern and elegant remedies.

DR. R. TILLEY: There is an inference, of course, even more manifest than appears in the title of the paper which was read to us, that we are indebted more or less to the homœopathists for the advance which we have made in the years gone by in the administration of medicines in a more palatable form. I claim, most emphatically, that such is not the case. The advance which we have made in this respect is to a large extent due to our chemists, and we are willing to accord to them the credit which belongs to them, but we are not willing to accord this credit to the homœopathists. Certainly we have all taken this subject into consideration; we have all given morphine, atropia, etc., when desired. All of us have, I am sure, been trying our best to render these medicines as palatable as possible. There, are, however, a few recommendations in the paper which I think would be most objectionable. For instance, I dare say few experienced men would dare to give iodide of potassium in large doses in capsules. I always recommend large quantities of water to be taken in connection with iodide of potassium. Then with reference to arsenic and antimony, we are told they can be administered in solutions in teaspoonful doses. We all know that, but I question whether it is desirable. I have always recommended the administration of arsenic in large quantities of water, and also tartar emetic. As to the administration of chloral in oil in capsules, I think the Doctor can claim the credit and have all the honor associated with it. There is one thing I would like to refer to, that is the administration of permanganate of potassium in capsule form. I have known it to be so administered in two or three cases, with very severe pain attending the administration, but I have never administered it in that way myself.

PROFESSOR J. H. ETHERIDGE: I think the paper is a very timely one, it is full of a great many good points, but I think there is an objection to the administration of concentrated remedies, especially these alkaloids, in gelatine covers and sugar-coated pills. It sometimes happens that after they have been administered they do not dissolve, and then we get a terrific effect by a half dozen dissolving at once, and in some cases they do not dissolve at all. I recall a case where it was decided to give a child thirty-five grains of quinine; seven capsules were administered and three of them passed the rectum untouched. They may not always promptly dissolve, and the consequence is we run a very great risk of injuring our patients.

DR. A. H. FOSTER: I am glad we have arrived at the point where our profession has learned to give less medicine, thus it is more palatable. Another thing, it is time that we began giving medicine more palatably by giving it in smaller doses, and we will often get a primary and not a secondary effect from our remedies, and will not get that terrible perturbation that we would if we gave large doses. Some of us have to deal with very delicate stomachs; that class of my practice which is more in the middle of the west side is more delicate than on the outskirts where they live more rough and ready, and I have to be a great deal more careful with these people who live more centrally, and make the doses smaller and more frequent to get the desired effect. But the point that I wish to make is, that we can and do have to study the palatability of our remedies. Another point is, that we have learned to simplify our remedies. I have had more than one physician prescribe in my family, and I always got along best with the one who didn't fire off shot-gun prescriptions. We very often fire shot-gun prescriptions, putting in cathartics and nervines into the same prescription; I would rather it would be one thing at a time and for a definite purpose.

PROFESSOR D. A. K. STEELE: There are a good many valuable points in this paper, and I think the criticism has pretty effectually squelched the objectionable ones. The author claims very valuable points from homœopathy; first, the palatability of their remedies; next, their safeness, that there is no danger in their remedies. That element of safety in prescribing we have applied in our own treatment, some of us, not from belief in homœopathy, but from unerring experience. After having almost killed a few patients in my early months of practice, with large doses and polyglot prescriptions, I came down to smaller doses and found them much safer. Now in reference to some of the objections that may be urged against palatable remedies; the objection to pills is their insolubility, the fact that they pass through the alimentary canals unchanged. I have repeatedly found them undissolved; found that in administering quinine in that form I did not get the desired result. I think the author made a mistake in giving too little credit to our pharmacists for the advances made in pharmacy in late years. The essential point of the paper, to my mind, is the fact that it impresses upon us the importance of giving less medicine and of giving it in a more palatable form.

DR. C. E. WEBSTER: There is a point we can learn from homœopathy, one lesson which, it seems, has not been touched upon, and that is that in the vast majority of ordinary acute diseases, careful nursing and absolutely inert remedies bring very creditable, if not the best, results; inert remedies which simply occupy the attention of the patient, will produce favorable results. This is the important and only lesson that can be learned from homœopathy.

DR. R. TILLEY: Dr. Foster seemed to imply that we get our tendency to give small doses from the homœopaths. I take the opportunity of saying that in the large hospitals in Paris and London, they have placed patients with acute affections side by side, and to the one they have given all the medicine they have thought necessary, and to the other they have given careful attention and nursing, with the suggestions a practical physician would give, and there has been practically no difference in the result in the two classes of cases. Experience has taught us to give in certain cases, or in many cases, simply a teaspoonful of water occasionally, and that is very important especially in children; a teaspoonful of water is frequently all that the child needs, although few people seem to realize the fact.

DR. DOERING: I object to crediting homœopathy with what science and chemistry have accomplished. Take the alkaloids, they have all been discovered in Germany, where homœopathy cannot live at all,—where they have not a single chair in any university. I think the paper would be much more valuable if all the reference to homœopathy had been omitted. Palatable medicines are simply the natural result of the advance of the science of medicine, chemistry and pharmacy.

PROFESSOR C. T. PARKES: I recall one instance which illustrates the fact that palatable medicines are not always innocent and harmless. I was called to make a post-mortem examination on a boy six years old. Upon opening the abdomen I found in the abdominal cavity a round mass, and upon examination of this round mass it was found to be a sugar-coated quinine pill. These were also found in the vermiform appendix and had ulcerated through into the abdominal cavity. So far as my opinion as to homœopathy is concerned, I wish to go on record as agreeing entirely with DR. DOERING.

DR. F. H. MARTIN closed the discussion by saying: In regard to the iodide of potassium, DR. TILLEY mentioned that he always gives iodide of potassium diluted in large quantities of water. When I was considering iodide of potash, I recalled the experience that I had myself in administering it, so I diluted the drug after getting it into the stomach; that is, I gave the patient water after he swallowed the capsule, and he experienced no trouble. In regard to permanganate of potash, it is not necessary to give it in capsules,—the oleate of manganese can be used by inunction, or the permanganate of potash be given in distilled water. In regard to the solubility of these pills, any of the best pills will dissolve in water in such a way that the remedies will become free in three minutes. If a capsule or pill of morphia be put in cold water, you can taste the morphia after it has been in the water not longer than two minutes, and you can readily see that by putting the pill into the stomach the gastric fluid, the temperature, and the muscular action, would soon cause its solution. In regard to the suggestion made by DR. WEBSTER, that the only lesson to be learned from homœopathy was the fact that a large per cent. would recover without remedies, I think I included that in my citing homœopathy as a lesson in general, for we all know from the success of homœopathy, if all their patients had died, or the majority of them had died, homœopathy would have gone out of existence immediately. But it was not the case, and it was taken for granted in the paper that the palatability and harmlessness of their remedies was the first great lesson of the evening and the paper. In regard to quinine pills, some time ago I believe that medicine was put up with sulphuric acid, and it lost strength and became almost inert, but now they put quinine up in excipients that will dissolve readily, and, also, loosely in flexible capsules.

The Society then adjourned.

---

CHICAGO MEDICAL SOCIETY.

